# Decreasing Sedentary Behavior: Effects on Academic Performance, Meta-Cognition, and Sleep

**DOI:** 10.3389/fnins.2017.00219

**Published:** 2017-05-09

**Authors:** June J. Pilcher, Drew M. Morris, Stewart A. Bryant, Paul A. Merritt, Hayley B. Feigl

**Affiliations:** Department of Psychology, Clemson UniversityClemson, SC, USA

**Keywords:** sedentary behavior, physical activity, performance, sleep, motivation, activity workstations

## Abstract

There is growing interest in using activity workstations as a method of increasing light physical activity in normally sedentary environments. The current study (*N* = 117) compared the effects of studying in college students while slowly pedaling a stationary bike with a desktop with studying at traditional desks across 10 weeks in an academic semester. The students were assigned to study either on the stationary bike or at a traditional desk located in the campus library for a minimum of 2 h a week. During the 10 weeks, the students studied for tests or worked on other required academic activities while working at their assigned desk. In addition, the participants completed a pre survey, weekly surveys, and a post survey. We found that although students studying at the traditional desks reported more ease of studying and more effective studying than those using the stationary bikes, the two groups performed equally well on tests in an introductory psychology course. Moreover, the students using the traditional desks reported a decrease in sleep quality later in the semester while those using the activity workstation reported stable levels of sleep quality. The current results indicate that activity workstations could be implemented in university settings to encourage light physical activity without negatively affecting academic performance while providing possible long-term health and well-being benefits. Furthermore, the results suggests that activity workstations could be a means of combating sedentary behavior in environments where individuals are expected to sit either while waiting (e.g., doctor's waiting rooms, airports) or when completing a necessary task (e.g., the workplace, educational settings).

## Introduction

Many studies have shown the positive effects of short-term regular moderate-to-vigorous physical activity on health, well-being, and performance (e.g., Warburton et al., 2006). Brief bouts of exercise; however, do not entirely counteract the potential negative effects of extended sedentary activity (Owen et al., 2009), indicating that too much sedentary behavior could be a separate health risk factor. Furthermore, many work and social settings encourage sedentary behavior resulting in inactive behavior increasingly replacing light-intensity physical activity during waking hours (Mansoubi et al., 2014). Previous research also suggests that trying to reverse this trend by replacing sedentary behavior with light-intensity activity could have positive metabolic benefits (Healy et al., 2008) and improve health (Torbeyns et al., 2014). Health researchers have also shown that light- or moderate-intensity activity (such as walking) is related to decreases in anxiety (Fox, 1999) and improved quality of life (Oka et al., 2000). As such, it is important to consider factors that may contribute to decreasing the amount of sedentary activity.

Physical activity decreases during adolescence (Stone et al., 1998) and with age (Mullineaux et al., 2001), resulting in many adults leading largely sedentary lives. Previous studies have found that persons in many types of work environments such as those working at desks or on computers (Hill et al., 2003) as well as many factory settings (Ishizaki et al., 2004) have higher levels of sedentary behavior. Moreover, sedentary behavior is related to a decrease in positive emotions (Hogan et al., 2015) while increased sitting time is related to increases in depression and anxiety (Rebar et al., 2014). In addition, sedentary behavior negatively affects metabolic health and decreases overall cognitive and brain health (Voss et al., 2014) whereas light physical activity such as pedaling a bicycle at a normal walking pace during two laboratory-based cognitive tasks for 35 min increases positive affect, motivation, and morale (Pilcher and Baker, 2016). As such, it is important to consider how to best incorporate activity throughout the day (Tudor-Locke and Schuna, 2012), particularly in sedentary settings where physical activity is limited (Smith et al., 2014). College campuses are one such setting where individuals do not participate in regular physical activity (Fountaine et al., 2011; Pengpid et al., 2015), resulting in many college students leading largely sedentary lives due to their time in class and time studying.

Only recently have researchers started to examine how work or educational settings can help encourage light physical activity in the place of normally sedentary activity and the resultant effects on performance, health, and well-being. One way to reduce sedentary behavior is to adapt normally indoor sedentary environments by providing activity workstations that encourage light physical activity while completing necessary or desired tasks. Recent research suggests that using activity workstations can increase energy expenditure (Tudor-Locke et al., 2014) and could have health benefits (Carson et al., 2014); however, there is limited research on the effects of implementing activity workstations into normally sedentary settings.

Little research has addressed the effects of light physical activity on daily functioning such as performance and meta-cognition (Rhodes et al., 2012) and the available literature provides conflicting results. Meta-cognition has been referred to as a self-awareness of mental activity and is important to consider with regards to the perceived effectiveness of studying. Some studies suggest activity workstations result in decreased performance on specific motor skills tasks (Straker et al., 2009), while other studies conclude that cognitive performance does not decrease while working at activity workstations (Cox et al., 2011; Carr et al., 2014; Pilcher and Baker, 2016). Research; however, suggests that the educational environment has an effect on college-level performance (Marchard et al., 2014). As such, additional research examining common types of cognitive tasks as well as the meta-cognitive effects while using activity workstations is needed to help determine if activity workstations can be implemented without adverse effects in many educational as well as work settings.

The primary purpose of the current experiment was to examine the effects of studying when riding a stationary bike with a desktop (FitDesk) in comparison to studying at a traditional desk on academic performance, meta-cognitive factors, and self-reported sleep. The meta-cognitive factors included student studying preferences and the benefits of active study habits. We hypothesize that using the FitDesk will not decrease academic performance. Due to the paucity of research, we could not generate testable hypotheses on the effects of using the FitDesk vs. a traditional desk on the meta-cognitive and sleep measures used in the current study.

## Materials and methods

### Participants

Participants were students in an introductory psychology class. One hundred and seventeen from a total of 249 students in the class completed the study (75 females, age 18.39 ± 0.94; 42 males, age 18.33 ± 0.72). Fifty-nine participants were block random assigned to the FitDesk condition (37 females, age 18.41 ± 0.96; 22 males, age 18.27 ± 0.63). The remaining 58 participants were assigned to the traditional desk condition (38 females, age 18.37 ± 0.94; 20 males, age 18.40 ± 0.82). The university's institutional review board approved the study. Inclusion criteria for this study included reporting being in good mental and physical health, and the physical ability to slowly pedal a bicycle for up to 2 h.

### Procedures

Students in an introductory psychology class were offered credit for required research participation as well as extra credit points to complete the study. The study was explained to the students in the class meeting following their first exam. Interested students signed the informed consent form which also gave experimenters permission to access their introductory psychology class grades. Researchers contacted the volunteers and asked them to complete a preliminary survey (see below). Participants were then assigned to the FitDesk study group or the traditional desk study group.

The FitDesks and traditional desks were located in the campus library providing access anytime the library was open. The FitDesk (Revo Innovations LLC, Antioch, TN) is a silent, stationary bike with a desktop. Users could work on a laptop, tablet, or other study materials while pedaling the bike. Participants were encouraged to peddle at a slow pace (similar in exertion to a normal walking pace) when using the FitDesk. All participants used their smart phones to scan QR codes posted in the room to indicate when they started and stopped studying. The time of logging in and logging out was automatically recorded. In addition, when logging out the participants indicated which FitDesk or traditional desk they used and briefly summarized what they worked on. The participants first used the FitDesks or traditional desks in the library for a 2-h acclimation period where they could do any type of activity (e.g., study for any class, on-line shopping). The participants were instructed to complete their acclimation period within 1 week after signing the consent form. After the acclimation week, participants were instructed to study only for their introductory psychology class for 2 h each week at their assigned desk type (FitDesk or traditional desk) for the 10 week study. Regular email reminders were sent informing the students of their number of minutes studying that week during the last 2 days of each week, to encourage all students to complete the full 2 h of study time for that week.

The participants completed weekly surveys (see below) that were initiated each Monday of the study and were completed by midnight on Tuesday. During the study, the participants completed their required in-class multiple-choice exams. At the end of the experiment, participants completed a final survey (see below). All surveys were administered electronically.

### Academic performance

Participants gave permission for the researchers to access their introductory psychology class grades on five exams, scores on a pre- and post-test (range of scores from 0 to 18), and their final grade in the course. The pre-test and exam 1 occurred before the onset of the study. Exams 2, 3, and 4 occurred during the study and were used to indicate academic performance during the study. Exam 5 and the post-test occurred after the conclusion of the study.

### Subjective measures

The preliminary survey contained one question on study habits, one question on class motivation, three questions on exercise, one question on physical health, and one question on sleep habits (see variable list in Table 1). These survey items were used to ensure that the two desk conditions were not different at the onset of the study. All questions on the preliminary survey were assessed using the same 5-point scale: almost never (1), less than half the time, about half the time, more than half the time, and almost always (5).

**Table 1:** **Academic Performance, survey items, and descriptive statistics**.

**Variable**	**Desk**	**Mean**	***SD***	**CI**	
				**Lower**	**Upper**
**ACADEMIC PERFORMANCE**					
Pre-test	FitDesk	7.12	2.65	−0.03	2.27
	Traditional desk	6.00	3.56		
Exam 1	FitDesk	86.03	11.89	−1.75	6.70
	Traditional desk	83.56	11.17		
Exam 2, 3, 4	FitDesk	85.80	8.90	−0.66	6.08
	Traditional desk	83.09	9.43		
Post-test	FitDesk	12.07	2.89	−1.36	0.75
	Traditional desk	12.37	2.24		
Final grade	FitDesk	85.33	9.57	−1.86	4.97
	Traditional desk	83.77	8.81		
**PRELIMINARY SURVEY**					
Overall, I have good study habits.	FitDesk	3.37	0.91	−0.30	0.39
	Traditional desk	3.33	0.98		
I am motivated to do well in my PSYC 2010 class.	FitDesk	4.66	0.48	−0.14	0.26
	Traditional desk	4.60	0.62		
I exercise daily.	FitDesk	3.10	1.26	−0.53	0.39
	Traditional desk	3.17	1.27		
I exercise on a regular basis (3–4 times a week).	FitDesk	3.78	1.34	−0.28	0.70
	Traditional desk	3.57	1.31		
I am motivated to exercise.	FitDesk	3.68	1.21	−0.32	0.61
	Traditional desk	3.53	1.34		
Overall, I am in good physical health.	FitDesk	4.39	0.72	−0.28	0.27
	Traditional desk	4.40	0.77		
Overall, I have good sleep habits.	FitDesk	3.63	1.08	0.09	0.89
	Traditional desk	3.14	1.08		
**WEEKLY SURVEY**					
Physical exertion^*^	FitDesk	2.02	1.07	0.33	1.52
	Traditional desk	1.10	2.05		
Motivated^*^	FitDesk	2.92	0.68	−0.54	−0.07
	Traditional desk	3.22	0.60		
Global morale	FitDesk	2.91	0.67	−0.43	0.03
	Traditional desk	3.11	0.57		
Global engagement	FitDesk	3.82	0.53	−0.36	0.02
	Traditional desk	4.00	0.51		
Committed^*^	FitDesk	3.69	0.65	−0.52	−0.08
	Traditional desk	3.99	0.55		
Completely absorbed^*^	FitDesk	2.86	0.90	−0.94	−0.32
	Traditional desk	3.49	0.77		
**FINAL SURVEY**					
How motivated did you feel when studying?	FitDesk	3.20	0.89	−0.56	0.11
	Traditional desk	3.43	0.94		
How focused were you while you were studying?	FitDesk	3.22	1.00	−0.62	0.06
	Traditional desk	3.50	0.86		
How successful did you feel at accomplishing your studying goals?^*^	FitDesk	3.22	1.02	−0.76	−0.07
	Traditional desk	3.64	0.85		
Did you feel that studying at the (type of desk) was effective?^*^	FitDesk	3.20	0.94	−0.84	−0.10
	Traditional desk	3.67	1.07		
Overall, how prepared were you for your tests in PSYC 2010?	FitDesk	3.83	0.67	−0.20	0.34
	Traditional desk	3.76	0.80		
Please estimate the amount of additional time you studied for PSYC 2010 per week during the semester OUTSIDE of the mandatory study periods in Library room 108.	FitDesk	102.14	167.72	−9.98	82.70
	Traditional desk	65.78	60.60		
Please estimate the amount of additional time you studied for PSYC 2010 in the week prior to each exam OUTSIDE of the mandatory study periods in Library room 108.	FitDesk	137.73	163.50	−5.52	86.67
	Traditional desk	97.16	68.95		
I feel that regularly studying for 2 h a week for PSYC 2010 helped me perform better in the class.	FitDesk	3.75	0.82	−0.62	−0.06
	Traditional desk	4.09	0.71		
I feel that studying on the (type of desk) helped me perform better in my PSYC 2010 class.^*^	FitDesk	3.34	0.86	−0.65	0.02
	Traditional desk	3.66	0.98		
I will use (type of desk) when studying or working in the future.^*^	FitDesk	3.07	1.34	−1.05	−0.12
	Traditional desk	3.66	1.21		
**SLEEP SURVEY**					
Sleep quality	FitDesk	3.56	0.70	−0.01	0.50
	Traditional desk	3.32	0.69		
Sleep quantity	FitDesk	7.89	1.01	−0.16	0.61
	Traditional desk	7.66	1.07		

Specific desk condition (FitDesk or Traditional desk) was inserted for “(type of desk)” in the surveys that the participants completed;

* *Significant difference between FitDesk and traditional desk users, exact p-values reported in text*.

The weekly survey (see variable list in Table 1) contained Borg's Rating of Perceived Exertion Scale (RPE; Borg, 1982, 1990). The RPE provides a subjective measure of exercise intensity and is related to physiological measures of physical exertion with validity coefficients ranging from 0.57 to 0.72 (Chen et al., 2002). The participants rated their level of perceived physical exertion while using their assigned desk type during the week on a scale from 0 (nothing at all) to 10 (very, very strong). The participants then filled out a single item question on motivation (5-point scale from very low to very high). A previous study using the FitDesk used this same single item motivation question and found that light physical activity when completing a complex cognitive task increased motivation (Pilcher and Baker, 2016). The five-item Morale Scale followed the motivation question and evaluated energy, drive, enthusiasm, eagerness, and morale (5-point scale from very low to very high) while studying during the week at their assigned desk type. The Morale Scale has been shown to have a Cronbach's alpha of 0.93 (Britt et al., 2013). A previous study using the FitDesk also found that light physical activity when completing a complex cognitive task increased morale using the Morale Scale (Pilcher and Baker, 2016). The weekly survey also contained six questions adapted from the Engagement Scale (e.g., sense of personal responsibility in studying, commitment to studying, completely absorbed in studying) to rate their studying at their assigned desk type using a 5-point Likert scale from strongly disagree to strongly agree (Britt et al., 2010). The Engagement Scale has been shown to be a predictor of performance in academic settings (Britt et al., 2010) and was used in a previous study with the FitDesk (Pilcher and Baker, 2016). The final portion of the weekly survey contained three questions about sleep habits (average time going to bed, average time waking up, and quality of sleep from very poor to very good).

The final survey (see variable list in Table 1) allowed participants to assess concepts that could be related to academic performance using a 5-point scale from not at all to very much. Concepts include study habits and feeling of motivation and focus experienced while studying. Questions on amount of time studying were also included in the final survey. Lastly, two questions on studying at the assigned desk type and whether the participant would use the desk type again when studying were assessed on a 5-point scale from strongly disagree to strongly agree.

### Statistical analyses

Data analysis was performed using the IBM SPSS statistical program (SPSS 22; SPSS Inc., Chicago, IL). A 2 × 3 mixed factors analysis of variance (ANOVA) was used to compare the two desk groups across the three exams. Independent samples *t*-tests were used to compare differences between the groups on all other measures except the sleep-related data. The data from the weekly surveys were averaged across the study. A global morale score was calculated by averaging the component questions on motivation, energy, enthusiasm, eagerness, and morale. A global engagement score was calculated by averaging the component questions on caring about studying, personal responsibility for studying, commitment to studying, importance of studying, and devotion to studying. Independent samples *t*-test were used to compare the desk groups for average sleep quantity and sleep quality across the 10-week period. Sleep quantity was calculated from the reported time the participant usually went to bed and the time they usually woke. As an additional analysis comparing the desk conditions, sleep data were collapsed across the first 5 weeks and last 5 weeks and compared. Two 2 (desk condition) × 2 (time, first 5 weeks and second 5 weeks) mixed factors ANOVAs were used to examine potential differences in self-reported sleep quantity and quality across the study. The Greenhouse-Geisser within-groups test is reported.

## Results

### Assigned study time

Participants in the Fitdesk condition spent an average of 1.92 (*SD* = 0.42) hours per week studying on the Fitdesk during the study. Similarly, participants in the traditional desk condition spent an average of 1.94 (*SD* = 0.38) hours per week studying on the traditional desk during the study. There was no significant difference in time studying between the two conditions.

### Academic performance

Descriptive statistics for all test scores are reported in Table 1. There were no significant differences in performance between the FitDesk and traditional desk groups on the pretest and exam 1. There were also no significant differences between the groups on the three exams during the study or on the posttest and final class grades.

### Subjective measures

Descriptive statistics for all subjective measures are reported in Table 1. Based on the preliminary survey, the participants in the FitDesk group were not significantly different from the traditional desk group on motivation to do well in the class, study habits, exercise routines, sleep habits, or physical health.

Weekly survey results showed that participants using the FitDesk reported greater physical exertion when studying than the participants using the traditional desk, *t*_(115)_ = 3.08, *p* = 0.003. It is important to note; however, that although the FitDesk users reported higher levels of perceived physical exertion, their average subjective rating was only “weak” physical exertion on the RPE. For comparison, the traditional desk users reported a subjective rating of “very weak” physical exertion. Traditional desk users reported feeling more motivated than those using the FitDesk, *t*_(115)_ = 2.56, *p* = 0.012. However, there was no significant difference in the global morale score or global engagement between groups. Traditional desk users also reported feeling more committed to studying, *t*_(115)_ = 2.67, *p* = 0.009, as well as more able to get completely absorbed in studying, *t*_(115)_ = 4.09, *p* < 0.001.

Final survey results showed no significant differences between groups in motivation, focus, feeling of preparedness for the tests, or feeling that the type of desk used improved performance in the class. There were also no significant differences between the two groups in the time spent studying for class outside of the prescribed desk times. Traditional desk users reported feeling that they were more successful in studying, *t*_(115)_ = 2.40, *p* = 0.018, and reported that studying at their desk was more effective, *t*_(115)_ = 2.52, *p* = 0.013, than the FitDesk users. Traditional desk users also reported that the 2-h weekly prescribed study time helped them perform better in class than FitDesk users did, *t*_(115)_ = 2.40, *p* = 0.018. Finally, both groups agreed that they would use their respective desks in the future when studying, though the traditional desk users reported higher levels than FitDesk users, *t*_(115)_ = 2.49, *p* = 0.014.

**Figure 1:**
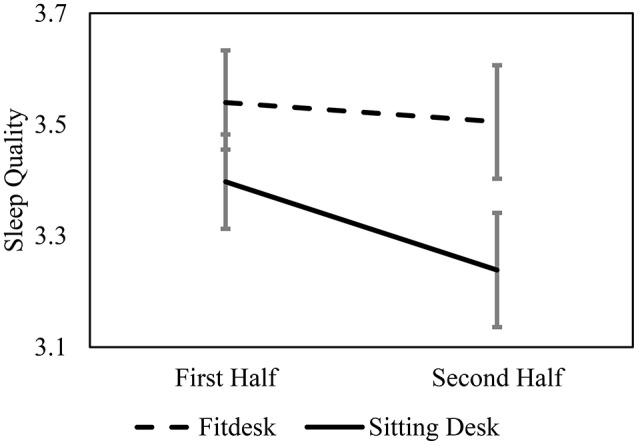
**Sleep Quality across the first 5 weeks and second 5 weeks of the study**. Values indicated as mean ± SE ranging from 1 (very poor) to 5 (very good).

Independent samples *t*-tests indicated that there was no significant difference in sleep quantity or sleep quality between groups when averaging across the 10 weeks of the study. However, sleep quality was decreasing toward the end of the semester in the traditional desk group (Figure 1). The 2 × 2 mixed factors ANOVA showed no main effects or interaction between time and desk condition on sleep quantity. In contrast, there was a main effect of desk condition on sleep quality. FitDesk users' sleep quality was stable between the first half and second half of the study while participants using a traditional desk reported a significant decrease in sleep quality from the first half to the second half of the study, *F*_(1, 57)_ = 7.82, *p* = 0.007, $\eta_{p}^{2}$ = 0.121 (Table 2). There was no main effect of time or significant interaction between groups on sleep quality.

**Table 2:** **Sleep Survey items and descriptive statistics**.

**Variable**	**Desk**	**Period**	**Mean**	***SD***
Sleep quality	FitDesk	First half	3.54	0.72
		Second half^*^	3.51	0.78
	Traditional desk	First half	3.40	0.65
		Second half^*^	3.24	0.78
Sleep quantity	FitDesk	First half	7.89	1.04
		Second half	7.88	1.06
	Traditional desk	First half	7.70	1.19
		Second half	7.62	1.12

First half: the first 5 weeks of the study, Second half: the second 5 weeks of the study;

* *Significant difference between FitDesk and traditional desk users, exact p-values reported in text*.

## Discussion

The current results indicate that individuals studied for equal periods of time on the FitDesk and traditional desks and those who studied at the FitDesk performed equally well on course exams as individuals who studied at a traditional desk. As expected, the FitDesk participants reported greater levels of physical exertion on the weekly surveys; however, their level of exertion was reported as “weak” on the RPE. In general, on the weekly and final surveys, the traditional desk participants reported being better able to study. This was seen in greater levels of motivation, commitment, and being absorbed on the weekly surveys as well as feeling more successful in studying and that studying at the traditional desk was more effective on the final survey. However, it is important to note that not all subjective measures were enhanced in the traditional desk group. The findings from the weekly surveys suggest that morale and engagement were the same in both groups and the findings from the final survey suggest there was no difference in motivation, focus, feeling of preparedness, or feeling that the type of desk improved performance. Finally, when collapsing across the 10-week study, there was no difference in either sleep quantity or sleep quality between the two conditions. When examining the last 5 weeks of the study; however, there was a decrease in sleep quality in the traditional desk participants but not the FitDesk participants.

The current findings provide support for our first hypothesis that studying at an activity workstation would not negatively impact academic performance. The FitDesk and traditional desk groups were equivalent in terms of performance in the course prior to the onset of the study and did not differ in the amount of time that they studied outside of the required desk times in the library. As such, the current data suggest that students could be less sedentary by increasing light physical activity when studying, a normally sedentary task, without negatively affecting academic performance. The current results also support conclusions from previous studies suggesting that activity workstations do not negatively impact cognitive performance (Carr et al., 2014; Pilcher and Baker, 2016).

It should be noted that the FitDesk group reported more time studying outside of the mandatory study periods in the library; however, this difference was insignificant due to a large degree of variability among the subjects. Because there is no significant difference between the FitDesk and traditional desk groups time studying outside of the library, it is difficult to interpret these results. It is possible that some students using the FitDesk felt that they were less attentive when studying and needed extra study time. Additional studies can be designed to more fully address this issue. Furthermore, other researchers have found a possible link between type of exercise and type of cognitive performance (Chang et al., 2012) indicating that the effects of using activity workstations may depend on the level of physical exertion and on specific task characteristics. The current study indicates that adding light physical activity when studying for 2 h a week is unlikely to have a strong positive or negative effect on course grades. Thus, adding activity workstations to a normally sedentary environment (e.g., a university library) could allow college students to become more active when studying and could be one method of improving health-related behaviors in young adults.

In general the traditional desk participants in the current study felt that they were more motivated and successful at studying. However, the traditional desk participants did not do better on the tests nor were they studying significantly more than the FitDesk participants, suggesting that their subjective responses could have been affected by the normalcy of studying at a traditional desk. As such, this difference could be another example of the disconnect seen in many college students between what is effective studying and what simply feels easier (Bjork et al., 2013; Dunlosky et al., 2013). Students often use an ease-of-acquisition heuristic when studying which results in an illusion of having learned the material because it feels easier (Kornell and Bjork, 2009), yet without having actually learned and understood the material. It is possible that using the FitDesk also creates a feeling of working harder; however, academic performance was not negatively impacted in the FitDesk group in the current study. Given the potential health and well-being benefits of participating in light physical activity while studying, it is important to determine the potential effects of using activity workstations and how they could be more fully implemented in university and other sedentary environments.

In spite of no changes in sleep quantity in response to desk condition, the sleep quality results in the current study indicate that light activity when studying could benefit sleep. This could be an important finding since the majority of college students report some level of sleep-related disturbances (Buboltz et al., 2001; Forquer et al., 2008) and sleep quality in college students is related to health and well-being (Pilcher et al., 1997). Furthermore, sleep could be related to blood pressure control (McCubbin et al., 2010) and parasympathetic activity (Walker et al., 2009), suggesting that stabilizing sleep quality could positively impact long-term health in college students. This is the first study that reports a possible link between changes in sleep quality with light physical activity when studying in college students. More research is needed to determine if increasing light physical activity in college students positively impacts sleep and other measures of health and well-being.

The current study has several limitations. One limitation was the participants used the FitDesk and traditional desk in the university library and were not monitored at all times. This allowed the participants greater accessibility to the study space and more naturalistic findings; however, it was possible for the participants to report that they were using a desk but not do so. The use of QR codes helped control for this natural limitation of a field-based study but future studies could be designed with more checks to better ensure that participants are using the desks when they scan the QR codes. A second limitation is the participants were asked to only study for their introductory psychology class during their study sessions; however, there were limits in how well we could control what the participants actually did when using the desks. To help control for this, we asked students to briefly summarize what they studied when they logged out and all students reported studying for their introductory psychology class. It seems that neither group would be more or less likely to follow the instructions and both groups did equally well on the course tests suggesting that this potential issue did not negatively affect the current results. Another possible limitation is that we did not attempt to monitor or limit participants based on current body weight or current levels of physical fitness. It must be noted, however, that we did include several questions about exercise and exercise habits in the preliminary studies and did not find any differences between the two groups. Future studies could be designed to control for body weight and other health-related issues. Finally, because the sleep measures were taken every week, we chose to use three questions as is done in many sleep research studies to limit the time and effort needed by the participants when completing the sleep logs (Pilcher et al., 2007; Odle-Dusseau et al., 2010). Future studies can be designed to use more detailed sleep surveys such as the Pittsburgh Sleep Quality Index.

## Conclusions

Our findings provide a unique comparison between the use of active and traditional workstations across an academic semester in a normally sedentary environment. FitDesk users experienced the potential benefits of increased light physical activity without sacrificing academic performance. Traditional desk users reported more subjective ease while studying, but this did not lead to better academic performance and may only highlight a misunderstanding associated with effective studying for many college students. These results suggest that implementing activity workstations on college campuses could have health and well-being benefits for the students without negatively affecting academic performance. Furthermore, research suggests that the appropriate use of cognitive control could lead to a less sedentary lifestyle (Buckley et al., 2014) and more positive sleep behaviors (Pilcher et al., 2015). Making active workstations available in normally sedentary environments could provide an option for a less sedentary lifestyle and more positive health behaviors. It is important to note that one explanation for some of the similarities between the FitDesk and traditional desk groups on some of the variables in the current study could be the limited required time studying at the assigned desk. For the purposes of this study, we choose 2 h to better fit into a variety of student study schedules. Future studies could examine the possible effects of a longer study period comparing the use of a traditional desk to the FitDesk. More research is also needed investigating how activity workstations can be implemented on college campuses and in other sedentary environments such as doctors' waiting rooms, waiting areas in airports, and sedentary job settings. The current results suggests that activity workstations may be a tool for combating sedentary lifestyle behavior in modern society without interrupting daily routine or negatively impacting performance.

## Ethics statement

Clemson University's institutional review board approved the study. All participants signed the informed consent form prior to the start of the study.

## Author contributions

JP conceived of the research question, designed the research protocol, oversaw data collection, organization, and analyses, and wrote the paper. DM assisted with data management and analyses and with writing the paper. SB assisted with the research protocol, and data collection, organization, and analyses and manuscript revision. PM assisted with data analyses and with writing the paper. HF assisted with the research protocol and data management and manuscript revision. All authors have approved the final version of the manuscript.

## Funding

Funding was provided by the Creative Inquiry and Undergraduate Research program at Clemson University.

### Conflict of interest statement

The authors declare that the research was conducted in the absence of any commercial or financial relationships that could be construed as a potential conflict of interest.
